# Relationship between Antioxidant Activity and Ligand Basicity in the Dipicolinate Series of Oxovanadium(IV) and Dioxovanadium(V) Complexes

**DOI:** 10.3390/ijms22189886

**Published:** 2021-09-13

**Authors:** Joanna Drzeżdżon, Marta Pawlak, Natalia Matyka, Artur Sikorski, Barbara Gawdzik, Dagmara Jacewicz

**Affiliations:** 1 Faculty of Chemistry, University of Gdańsk, Wita Stwosza 63, 80-308 Gdańsk, Poland; joanna.drzezdzon@ug.edu.pl (J.D.); marta.pawlak0812@gmail.com (M.P.); natmatyla@gmail.com (N.M.); artur.sikorski@ug.edu.pl (A.S.); dagmara.jacewicz@ug.edu.pl (D.J.); 2 Institute of Chemistry, Jan Kochanowski University, Uniwersytecka 7, 25-406 Kielce, Poland

**Keywords:** oxovanadium(IV) complexes, 2-phenylpyridine, superoxide anion, antioxidant activity

## Abstract

Oxidative stress plays an important role in the pathogenesis of many serious diseases, including cancer, atherosclerosis, coronary artery disease, Parkinson’s disease, Alzheimer’s disease, stroke and myocardial infarction. In the body’s natural biochemical processes, harmful free radicals are formed, which can be removed with the help of appropriate enzymes, a balanced diet or the supply of synthetic antioxidant substances such as flavonoids, vitamins or anthocyanins to the body. Due to the growing demand for antioxidant substances, new complex compounds of transition metal ions with potential antioxidant activity are constantly being sought. In this study, four oxovanadium(IV) and dioxovanadium(V) dipicolinate (dipic) complexes with 1,10-phenanthroline (phen), 2,2′-bipyridyl (bipy) and the protonated form of 2-phenylpyridine (2-phephyH): (1) [VO(dipic)(H_2_O)_2_]·2 H_2_O, (2) [VO(dipic)(phen)]·3 H_2_O, (3) [VO(dipic)(bipy)]·H_2_O and (4) [VOO(dipic)](2-phepyH)·H_2_O were synthesized including one new complex, so far unknown and not described in the literature, i.e., [VOO(dipic)](2-phepyH)·H_2_O. The oxovanadium(IV) dipicolinate complexes with 1,10-phenanthroline and 2,2′-bipyridyl have been characterized by several physicochemical methods: NMR, MALDI-TOF-MS, IR, but new complex [VOO(dipic)](2-phepyH)·H_2_O has been examined by XRD to confirm its structure. The antioxidant activities of four complexes have been examined by the nitrotetrazolium blue (NBT) method towards superoxide anion. All complexes exhibit high reactivity with superoxide anion and [VOO(dipic)](2-phepyH)·H_2_O has higher antioxidant activity than L-ascorbic acid. Our studies confirmed that high basicity of the auxiliary ligand increases the reactivity of the complex with the superoxide radical.

## 1. Introduction

The human body has many defense mechanisms that neutralize the harmful effects of reactive oxygen species. Antioxidants play an important role in reducing oxidative damage in the human body [1,2,3,4,5]. These are compounds which, even at a very low concentration, compared to the substrate, can delay or prevent its oxidation. Antioxidants can be divided into two groups. The first are antioxidants that interrupt radical reactions by donating hydrogen atoms or electrons to radicals, which leads to the formation of compounds with greater stability. Such compounds include: tocopherols [6,7], phenols [8], hydroquinones [9]. The second group includes substances whose action is synergistic. They are capable of scavenging oxygen and chelating ions involved in the formation of radicals [10].

According to the reaction mechanism, methods for measuring antioxidant capacity can be divided into methods based on hydrogen atom transfer (HAT), electron transfer (ET), and both. In the methods based on the transfer of the hydrogen atom, the result depends on the dissociation energy of the bond and the ionization potential in the group that is the donor of the hydrogen atom. HAT reactions are usually fast and independent of the solvent and the pH of the environment. Electron transfer reactions depend on the ionization potential of the active functional group in the antioxidant molecule, and therefore also depend on the pH. The value of the ionization potential decreases with increasing pH, as the electron-donor capacity increases [11,12,13,14]. Antioxidants have multiple effects: inter alia, they prevent the formation of oxidants, i.e., free radicals, inhibit the initiation of the oxidation process of metals such as cadmium, mercury, copper and lead, which is associated with supporting the immune system, or they intercept the reactive oxidants—oxidants and inhibit reactions chain—which threaten the formation of radicals [15].

Recently, there has been increasing interest in complex compounds containing d-block metal ions with organic ligands exhibiting antioxidant properties. The literature describes the antioxidant properties of copper(II) complex compounds with heterocyclic and polycarboxylate ligands: e.g., [Cu(ida)(phen)(H_2_O)] ∙ 2 H_2_O; [Cu(dipic)(4-pic)]_n_, [Cu(oda)(4-pic)H_2_O] ∙ 2 H_2_O, [Cu(oda)(bipy)(H_2_O)] ∙ 4 H_2_O (ida denotes iminodiacetate, oda = oxydiacetate, 4-pic = 4-picolinate, phen = 1,10-phenanthroline, bipy = 2,2′-bipyridyl). Studies have shown that copper(II) coordination compounds are antioxidants, but unfortunately in a concentration that is about 450–1550 times greater than superoxide dismutase [16]. Therefore, our interests focused on the antioxidant properties of oxovanadium(IV) and dioxovanadium(V) complex compounds. The methods of synthesis, characterization and biological properties of dioxovanadium(V) OH-substituted dipicolinate complexes have been previously described in the literature [17]. These complexes exhibit insulin-like activity. The Crans group conducted research on Cl-substituted dipicolinate complexes of vanadium(III, IV, V) and they confirmed that these compounds cause anti-diabetic effect on rats [18]. Studies have confirmed that the oxygenation state of vanadium has an impact on the insulin-like properties of complexes [18]. The antioxidant properties of the vanadium complexes can be directed towards the two-electron transfer reactions then their toxicity is lowered [19,20].

In this study, four oxovanadium(IV) and dioxovanadium(V) dipicolinate (dipic) complexes with 1,10-phenanthroline (phen), 2,2′-bipyridyl (bipy) and the protonated form of 2-phenylpyridine (2-phephyH): (1) [VO(dipic)(H_2_O)_2_]·2 H_2_O, (2) [VO(dipic)(phen)]·3 H_2_O, (3) [VO(dipic)(bipy)]·H_2_O and (4) [VOO(dipic)](2-phepyH)·H_2_O were synthesized including one new complex, so far unknown and not described in the literature, i.e., [VOO(dipic)](2-phepyH)·H_2_O. The oxovanadium(IV) dipicolinate complexes with 1,10-phenanthroline and 2,2′-bipyridyl have been characterized by several physicochemical methods: NMR, MALDI-TOF-MS, IR, but new complex [VOO(dipic)](2-phepyH)·H_2_O has been examined by XRD to confirm its structure. All four complexes have been tested towards antioxidant activities by the nitrotetrazolium blue (NBT) method against the superoxide anion. The purpose of the studies was the examination of the impact of heterocyclic ligands on the reactivity of the complex with superoxide anion.

## 2. Results and Discussion

**Figure 1:**
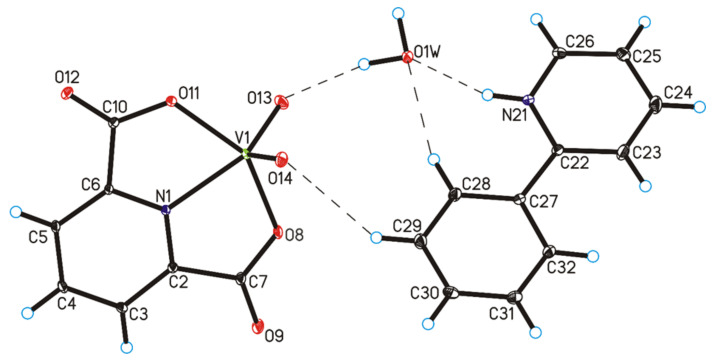
Molecular structure of [VOO(dipic)](2-phepyH)·H_2_O, showing the atom-labelling scheme (hydrogen bonds are represented by dashed lines).

**Figure 2:**
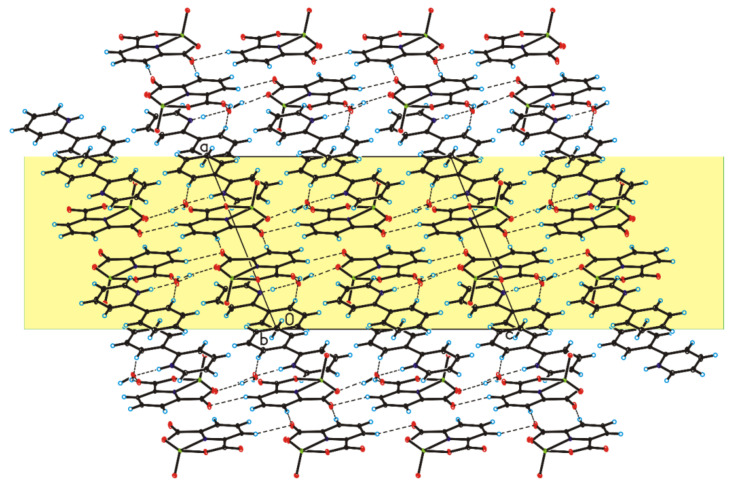
Crystal packing of [VOO(dipic)](2-phepyH)·H_2_O viewed along *b*-axis.

The four complex compounds were synthesized: (1) [VO(dipic)(H_2_O)_2_]·2 H_2_O, (2) [VO(dipic)(phen)]·3 H_2_O, (3) [VO(dipic)(bipy)]·H_2_O and (4) [VOO(dipic)](2-phepyH)·H_2_O (Figure 1). The complexes (1)-(3) are known in the literature [21,22]. [VOO(dipic)](2-phepyH)·H_2_O is a new complex compound previously not described in the literature. Single-crystal XRD measurements show that [VOO(dipic)](2-phepyH)·H_2_O crystallize in the monoclinic *P*2_1_/c space group with one 2-phenylpyridinium cation, one dioxo-(pyridine-2,6-dicarboxylato)-vanadium(V) anion and one water molecule in the asymmetric unit (Figure 1, Table 1). The bond lengths and angles characterizing the geometry of the dioxo-(pyridine-2,6-dicarboxylato)-vanadium(V) cation are similar to those observed in the other crystal structures containing this ion [23,24]. In the crystal of the title compound, ions and water molecules are linked via N–H···O_(water)_, O_(water)_–H···O and C–H···O hydrogen bonds to form blocks along *b*-axis (Figure 2—highlighted in yellow, Table 2). The neighbouring blocks are linked via π–π interactions between 2-phenylpyridinium cations to form a 3D framework structure.

The results of elemental analysis of [VO(dipic)(H_2_O)_2_]·2 H_2_O, [VO(dipic)(phen)]·3 H_2_O, [VO(dipic)(bipy)]·H_2_O are as follows: [VO(dipic)(H_2_O)_2_]·2 H_2_O showed 27.64% C, 3.60% H, and 4.70% N; and analysis calculations included 27.63% C, 2.96% H, and 4.61% N. Sample of [VO(dipic)(phen)]·3 H_2_O exhibited 48.94% C, 3.68% H, and 8.89% N; analysis calculations showed 48.93% C, 3.65% H, and 9.01% N. Sample of [VO(dipic)(bipy)]·H_2_O showed 49.99% C, 3.35% H, and 10.29% N; analysis calculations included 50.25% C, 3.20% H, 10.34% N. IR analysis confirmed the structure of the following complexes: [VO(dipic)(H_2_O)_2_]·2 H_2_O, [VO(dipic)(phen)]·3 H_2_O, [VO(dipic)(bipy)]·H_2_O (Table 3, Figures S1–S3).

MALDI-TOF-MS spectra allowed to identify the fragmentation of the studied complexes. For [VO(dipic)(H_2_O)_2_]·2 H_2_O: [M+H]^+^ 303.94 m/z; [M–2H_2_O+H]^+^ 268.11 m/z; [M–4H_2_O+H]^+^ 231.89 m/z; for [VO(dipic)(phen)]·3 H_2_O: [M+H]^+^ 407.00 m/z; [M–H_2_O+H]^+^ 388.97 m/z; and for [VO(dipic)(bipy)]·H_2_O: [M+H]^+^ 303.94 m/z; [M–2H_2_O+H]^+^ 268.11 m/z; [M–4H_2_O+H]^+^ 231.89 m/z (Figures S4–S9).

Due to the too high water content in the coordination compounds [VO(dipic)(H_2_O)_2_]·2 H_2_O and [VO(dipic)(phen)]·3 H_2_O it was impossible to obtain good quality NMR spectra for these compounds. Only for the complex [VO(dipic)(bipy)]·H_2_O ^1^H NMR and ^13^C NMR spectra was obtained in good quality. The results of NMR showed that the peaks observed on ^1^H NMR spectrum: 7.46, 7.95, 8.40, 8.70 ppm correspond to the peaks protons from dipic and the peak at 3.38 ppm means H_2_O presence. ^13^C NMR spectrum showed the peaks at: 155.68, 149.76, 137.81, 124.69, 120.92 ppm which correspond to C atoms from dipic (Figures S10 and S11).

**Figure 3:**
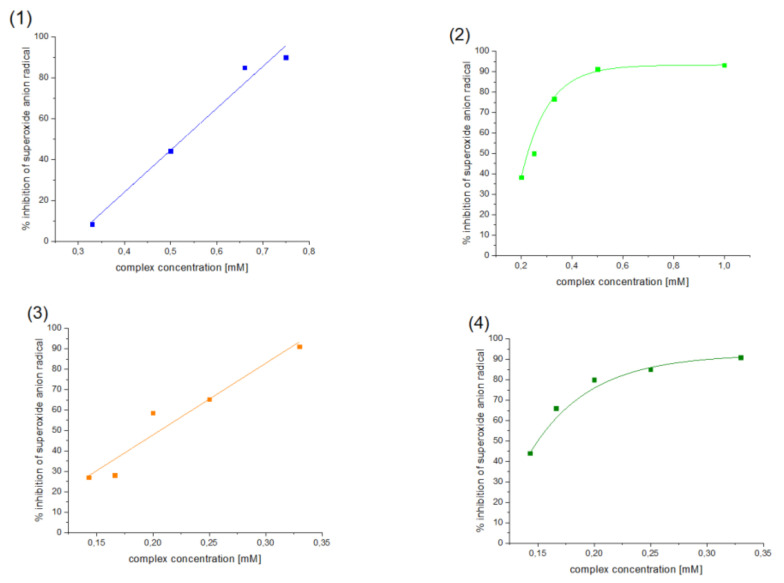
Inhibition of superoxide anion radical by (**1**) [VO(dipic)(H_2_O)_2_]·2 H_2_O, (**2**) [VO(dipic)(phen)]·3 H_2_O, (**3**) [VO(dipic)(bipy)]·H_2_O and (**4**) [VOO(dipic)](2-phepyH)·H_2_O.

Antioxidant properties of [VO(dipic)(H_2_O)_2_]·2 H_2_O, [VO(dipic)(phen)]·3 H_2_O, [VO(dipic)(bipy)]·H_2_O and [VOO(dipic)](2-phepyH)·H_2_O have been investigated towards superoxide anion radical by NBT method. The obtained results were presented in Figure 3.

**Figure 4:**
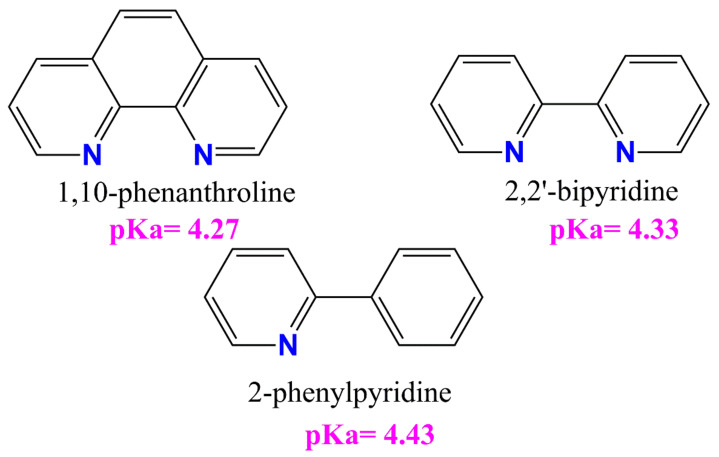
The auxiliary heterocyclic ligand used in the synthesis of dipicolinate oxo- and dioxo- vanadium(IV) complexes [25,26,27].

All tested compounds showed antioxidant properties, and the best antioxidant (even better than L-ascorbic acid) turned out to be the complex [VOO(dipic)](2-phepyH)·H_2_O, the calculated equivalent towards L-ascorbic acid for it is 0.68. On the other hand, the complexes [VO(dipic)(bipy)]·H_2_O and [VO(dipic)(phen)]·3 H_2_O exhibit antioxidant properties similar to L-ascorbic acid, when their equivalents are 0.936 and 1.032, respectively. The weakest antioxidant against the superoxide radical anion among the synthesized coordination compounds turned out to be [VO(dipic)(H_2_O)_2_]·2 H_2_O, whose equivalent is 2.359. The results of the research allowed us to draw a conclusion that the greater the basicity of the auxiliary ligand, the better the antioxidant properties against the superoxide anion radical (Figure 4). 2-Phenylpyridine is the strongest basic and thus the complex which contains this ligand in the coordination sphere of vanadium(IV) shows the strongest antioxidant properties, whereas 1,10-phenanthroline is the least basic and thus the complex which contains this ligand in the coordination sphere of vanadium(IV) shows the weakest antioxidant properties, of course, except for the complex which does not contain any auxiliary heterocyclic ligand at all ([VO(dipic)(H_2_O)_2_]·2 H_2_O).

## 3. Materials and Methods

To synthesize [VO(dipic)(H_2_O)_2_]·2 H_2_O, 2.65 g of vanadyl acetylacetonate was mixed with 1.67 g of dipicolinic acid (H_2_dipic) and 50 mL of distilled water was added. The result was the green solution. The resulting solution was heated to reflux for about 30 min. After cooling, blue crystals of the complex compound [VO(dipic)(H_2_O)_2_]·2 H_2_O were precipitated [22]. IR (KBr, cm^−1^): 3618, 3571, 3087, 2035, 1955, 1890, 1666, 1597, 1470, 1435, 1352, 1263, 1177, 1149, 1074, 1034, 984, 928, 853, 833, 767, 684, 595, 452.

To synthesize [VO(dipic)(phen)]·3 H_2_O, 2.65 g (0.01 mol) of vanadyl acetylacetonate was mixed with 1.67 g (0.01 mol) of dipicolinic acid and 1.98 g (0.01 mol) of 1,10-phenanthroline monohydrate and all reagents were dissolved in 50 mL of distilled water. The result was a brownish-orange solution. The resulting solution was heated until the solution changed color for about 3 min. After cooling, a red-brown crystals of [VO(dipic)(phen)]·3 H_2_O were obtained [22]. IR (KBr, cm^−1^ ): 3428, 3069, 3047, 3003, 1978, 1953, 1694, 1666, 1649, 1627, 1596, 1517, 1493, 1467, 1422, 1319, 1225, 1158, 1106, 1081, 1037, 980, 909, 868, 847, 800, 769, 741, 723, 693, 680, 644, 594, 444, 417.

To synthesize [VO(dipic)(bipy)]·H_2_O, 2.65 g (0.01 mol) of vanadyl acetylacetonate was mixed with 1.67 g (0.01 mol) of dipicolinic acid and 1.56 g (0.01 mol) of 2,2′-bipyridyl (bipy). All reagents were dissolved in 50 mL of distilled water. The result was a yellow—green solution was obtained. The resulting solution was heated until the solution changed color for about 4 min. Upon cooling the solution, a dark brown precipitate appeared, which was a complex [VO(dipic)(bipy)]·H_2_O [22]. IR (KBr, cm^−1^): 3537, 3422, 3278, 3104, 3075, 3054, 1946, 1917, 1681, 1649, 1598, 1572, 1496, 1476, 1444, 1427, 1356, 1335, 1319, 1245, 1185, 1166, 1086, 1040, 1023, 977, 914, 861, 824, 775, 743, 685, 651, 632, 617, 596, 442. ^1^H NMR (298 K) 500.13 MHz (DMSO-d6, δ): 8.70 ppm, 8.40 ppm, 7.95 ppm, 7.46 ppm, 3.38 ppm (H_2_O), 2.51 ppm (DMSO). ^13^C NMR (298 K) 125.76 MHz (DMSO-d6, δ): 155.68, 149.76, 137.81, 124.69, 120.92 ppm, 40.19 ppm.

The complex [VOO(dipic)](2-phepyH)·H_2_O synthesis was carried out according to the following procedure: vanadyl acetylacetonate (2.65 g, 0.01 mol), dipicolinic acid (1.67 g, 0.01 mol) and 2.86 mL (0.02 mol, density 1.086 g·mL^−1^) of 2-phenylpyridine were mixed with 50 mL of water. It was heated to reflux until the solution changed color. After cooling, brown crystals of the complex [VOO(dipic)](2-phepyH)·H_2_O were formed.

All materials used in this work have been purchased from Merck.

Diffraction data were collected on an Oxford Diffraction Gemini R ULTRA Ruby CCD diffractometer (T = 295(2) K, MoK_α_ (λ = 0.71073 Å), CrysAlis RED software (ver. 1.171.41.16a) [28]. The structures were refined and solved using the SHELX package (ver. 2017/1) [29]. H-atoms from water molecules were located on a difference Fourier map and refined with restraints (DFIX command) with *d_(_*_O–H)_ = 0.95 Å and U_iso_(H) = 1.5U_eq_(O), while H–atoms bound to C–atoms were placed geometrically and refined using a riding model with *d*_(C–H)_ = 0.93 Å and U_iso_(H) = 1.2U_eq_(C). All interactions were identified using the PLATON program (ver. 181115) [30]. The ORTEPII [31], PLUTO-78 [32] and Mercury (ver. 2020.2.0) [33] programs were used to prepare the molecular graphics.

Full crystallographic details for the title compound have been deposited in the Cambridge Crystallographic Data Center (deposition No. CCDC 2087422) and they may be obtained from http://www.ccdc.cam.ac.uk (accessed on 6 June 2021), e-mail: deposit@ccdc.cam.ac.uk or The Director, CCDC, 12 Union Road, Cambridge, CB2 1EZ, UK.

Elemental analysis of the complex compounds was performed using the Vario EL Cube analyzer and the percentage of carbon, nitrogen and hydrogen in the tested compound was determined.

MALDI-TOF-MS spectra were obtained using a Bruker Biflex spectrometer III. The following matrices were used: 2,5-hydroxybenzoic acid and α- cyano-4-hydroxycinnamic acid.

The IR spectra were recorded on a KBr tablet using a BRUKER IFS 66 spectrophotometer. Measurements were carried out in the range from 4000 cm^−1^ to 650 cm^−1^.

The NMR spectra were recorded on a Bruker Avance III 500 instrument. DMSO-d6 was used as a solvent.

The NBT test: a solution containing the superoxide anion radical was prepared. Initially, 6.5 mg of KO_2_ and 90 mg of 18-crown-6 were dissolved in 50 mL of DMSO. The solution was then placed under ultrasound for 7 min. In the next step, an NBT solution with a concentration of 1 mg/mL was prepared. The next step was to prepare solutions of complex compounds in DMSO. Depending on the solubility of a given complex compound, a 1mM stock solution was prepared, and then subsequent solutions of the complex compounds were prepared by diluting the stock solution. The measurement samples were prepared by mixing 1.5 mL of the radical solution in DMSO, 0.5 mL of the complex compound solution, and 0.1 mL of NBT. Control samples contained 1.5 mL of superoxide anion solution, 0.5 mL of DMSO and 0.1 mL of NBT solution. The reaction of the superoxide anion radical with NBT was monitored spectrophotometrically at a wavelength of 560 nm. The reference substance was L-ascorbic acid. Measurement of absorbance was carried out approximately 30 min after mixing the reactants. Measurement of absorbance during NBT testing was carried out on a Perkin-Elmer Lambda 45 spectrophotometer. The apparatus used is characterized by a Peltier system with a reading accuracy of 1 nm and a gap width of 1 nm at a scanning speed of 120.00 nm min^−1^ combined with a thermostatic system.

## 4. Conclusions

The structure of the new complex [VOO(dipic)](2-phepyH)·H_2_O was described for the first time. Moreover, the performed spectroscopic analyzes and elemental analysis confirmed the composition of the obtained series of dipicolinate oxovanadium(IV) coordination compounds. The conducted research confirmed that the synthesized complex compounds (1) [VO(dipic)(H_2_O)_2_]·2 H_2_O, (2) [VO(dipic)(phen)]·3 H_2_O, (3) [VO(dipic)(bipy)]·H_2_O and (4) [VOO(dipic)](2-phepyH)·H_2_O exhibit antioxidant properties against superoxide anion radical. The best reactivity towards the superoxide radical of the synthesized oxo- and dioxo- vanadium(IV) complex compounds showed [VOO(dipic)](2-phepyH)·H_2_O, while the weakest antioxidant with of the synthesized coordination compounds turned out to be [VO(dipic)(H_2_O)_2_]·2 H_2_O. Moreover, the results of this study showed that the high basicity of the auxiliary ligand increases the reactivity of the complex with the superoxide radical. The studied group of compounds may be further investigated in the future for the potential use of these complexes as superoxide dismutase mimetics.

## Figures and Tables

**Table 1:** Crystal data and structure refinement for [VOO(dipic)](2-phepyH)·H_2_O.

Chemical Formula	C_18_H_15_N_2_O_7_V
Formula weight/g·mol^−1^	422.26
Crystal system	monoclinic
Space group	*P*2_1_/c
*a*/Å	11.7061(9)
*b*/Å	10.6882(5)
*c*/Å	15.3271(11)
*α*/°	90
*β*/°	111.967(8)
*γ*/°	90
*V*/Å^3^	1778.5(2)
*Z*	2
*T*/K	295(2)
*λ*_Mo_/Å	0.71073
*ρ_cal_*_c_/g·cm^–3^	1.577
*F(000)*	864
µ/mm^−1^	0.603
*θ* range/°	3.35–25.00
Completeness *θ*/%	99.8
Reflections collected	11911
Reflections unique	3121 [R_int_ = 0.0525]
Data/restraints/parameters	3121/3/262
Goodness of fit on *F^2^*	1.058
Final R_1_ value (*I* > 2σ(*I*))	0.0468
Final *w*R_2_ value (*I* > 2σ(*I*))	0.0992
Final R_1_ value (all data)	0.0655
Final *w*R_2_ value (all data)	0.1069
CCDC number	2087422

**Table 2:** Hydrogen bonds geometry in the crystal of [VOO(dipic)](2-phepyH)·H_2_O.

D–H···A	d(D–H) [Å]	d(H···A) [Å]	d(D⋯A) (Å)	∠D–H⋯A (°)
O1W–H1WA···O12 ^i^	0.95(2)	1.91(2)	2.811(3)	158(4)
O1W–H1WB···O13	0.95(2)	1.91(2)	2.826(3)	161(4)
N21–H21···O1W	0.87(3)	1.86(3)	2.699(4)	161(4)
C3–H3···O9 ^ii^	0.93	2.40	3.330(4)	177
C5–H5···O9 ^iii^	0.93	2.38	3.250(4)	157
C26–H26···O11 ^i^	0.93	2.35	3.135(4)	142
C28–H28···O1W	0.93	2.44	3.345(4)	165
C29–H29···O14	0.93	2.58	3.389(5)	145

Symmetry code: (^i^) x,½ − y, ½ + z; (^ii^) 1 − x,2 − y,1 − z; (^iii^) x,3/2 − y, − ½ + z.

**Table 3:** IR data of [VO(dipic)(H_2_O)_2_]·2 H_2_O, [VO(dipic)(phen)]·3 H_2_O, [VO(dipic)(bipy)]·H_2_O.

	[VO(dipic)(H_2_O)_2_]·2 H_2_O	[VO(dipic)(phen)]·3 H_2_O	[VO(dipic)(bipy)]·H_2_O
V=O stretching frequency	983 cm^−1^	980 cm^−1^	977 cm^−1^
*v*(COO) of dipic [18]	1352 cm^−1^	1319 cm^−1^	1335 cm^−1^
	1665 cm^−1^	1666 cm^−1^	1649 cm^−1^
*v*(OH) [18]	3571 cm^−1^	3427 cm^−1^	3536 cm^−1^
stretching vibration of the V-N	452 cm^−1^	416 cm^−1^	442 cm^−1^

## Supplementary Materials

The following are available online at https://www.mdpi.com/article/10.3390/ijms22189886/s1.
