# Supplementary material for: Relationship between Antioxidant Activity and Ligand Basicity in the Dipicolinate Series of Oxovanadium(IV) and Dioxovanadium(V) Complexes
Source: Int J Mol Sci. 2021 Sep 13;22(18):9886. doi: 10.3390/ijms22189886 (PMC8467204; doi:10.3390/ijms22189886)
Supplement: Supplementary file 1 [file ijms-22-09886-s001.zip › ijms-1338236-supplementary pdf.pdf]

Supplementary Materials

Relationship between antioxidant activity and ligand basicity in the dipicolinate series of oxovanadium(IV) and dioxovanadium(V) complexes

Joanna Drzeżdżon[1], Marta Pawlak[1], Natalia Matyka[1], Artur Sikorski[1], Barbara Gawdzik[2,*] and Dagmara Jacewicz[1]

[1] Faculty of Chemistry, University of Gdańsk, Wita Stwosza 63, 80-308 Gdańsk, Poland; joanna.drzezdzon@ug.edu.pl (J.D.); marta.pawlak0812@gmail.com (M.P.); natmatyla@gmail.com (N.M.); artur.sikorski@ug.edu.pl (A.S.); dagmara.jacewicz@ug.edu.pl (D.J.);
[2] Institute of Chemistry, Jan Kochanowski University, Świętokrzyska 15 G, 25-406 Kielce, Poland; b.gawdzik@ujk.edu.pl
* Correspondence: b.gawdzik@ujk.edu.pl; Tel.: +48 41 349 70 16

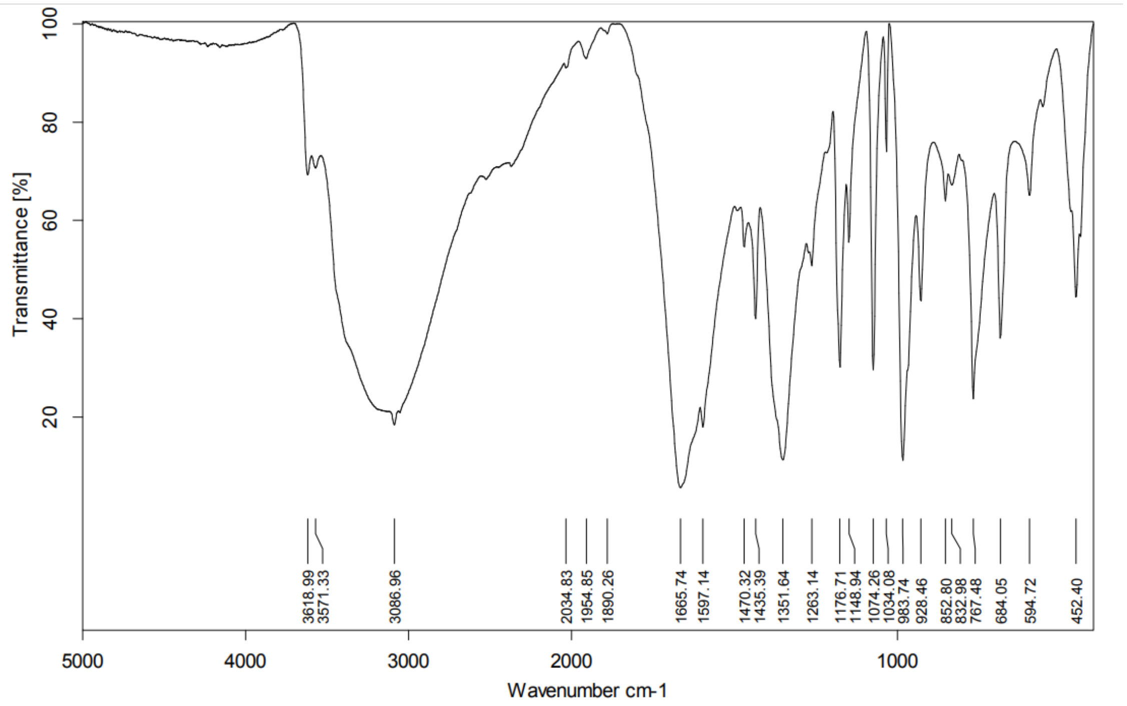

Figure S1. IR spectrum of $[VO(dipic)(H_2O)_2] \cdot 2\ H_2O$.

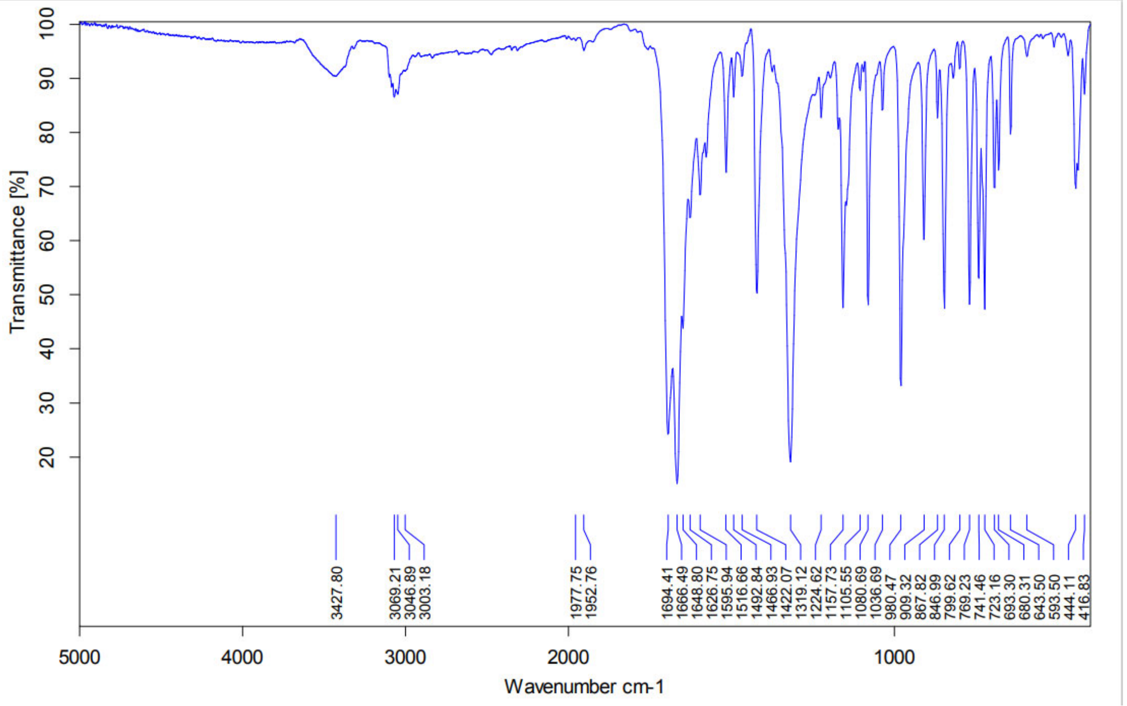

Figure S2. IR spectrum of $[VO(dipic)(phen)] \cdot 3\ H_2O$.

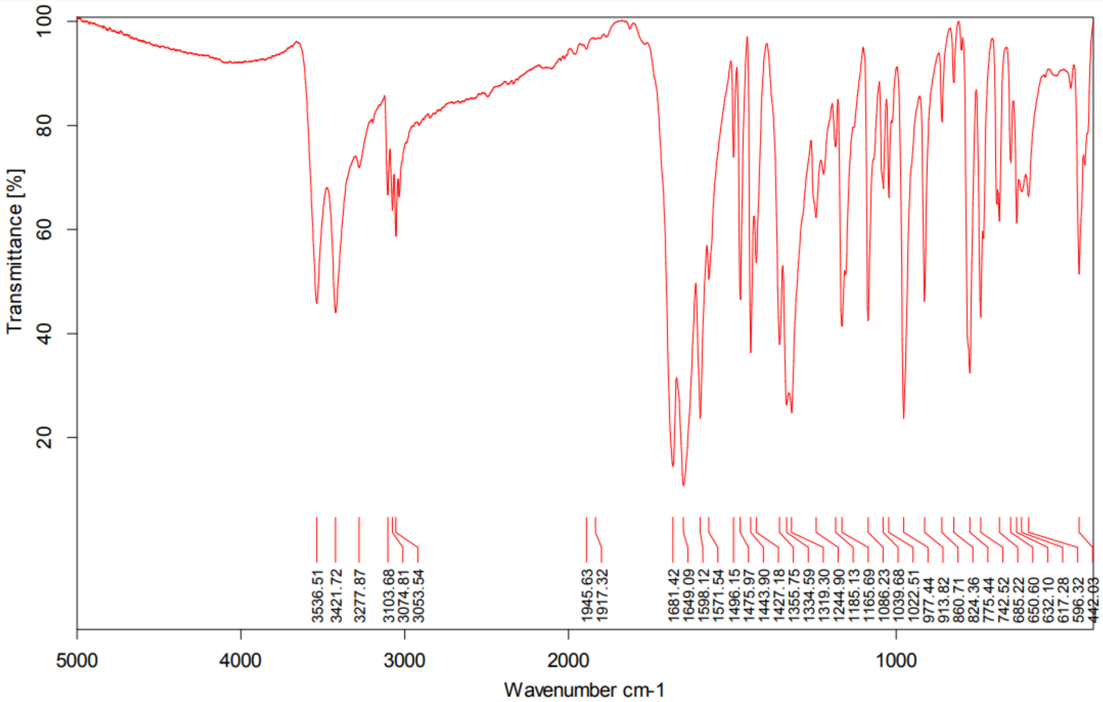

Figure S3. IR spectrum of $[VO(dipic)(bipy)] \cdot H_2O$.

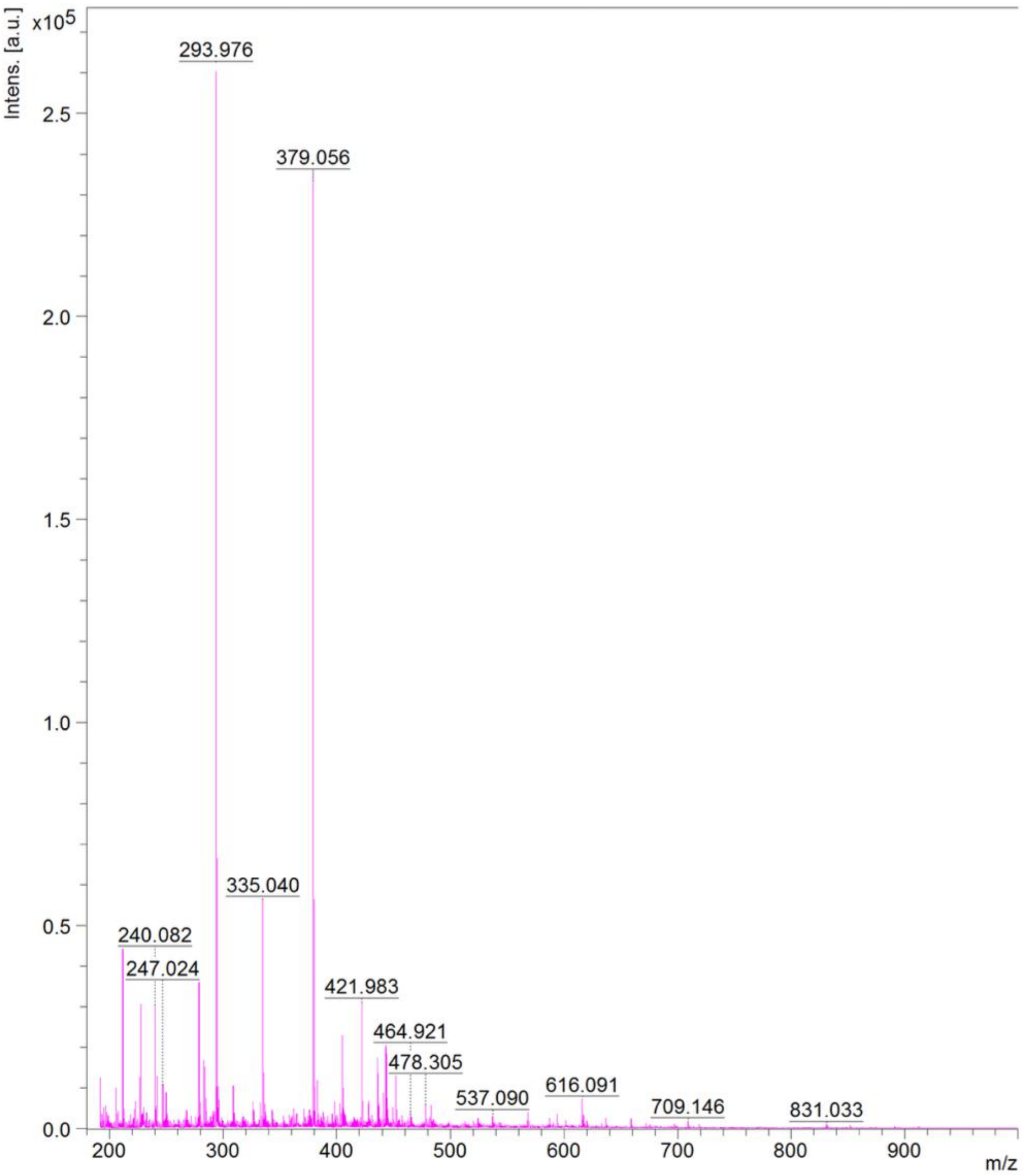

Figure S4. MALDI-TOF-MS spectrum of $[VO(dipic)(H_2O)_2] \cdot 2\ H_2O$ (CCA matrix).

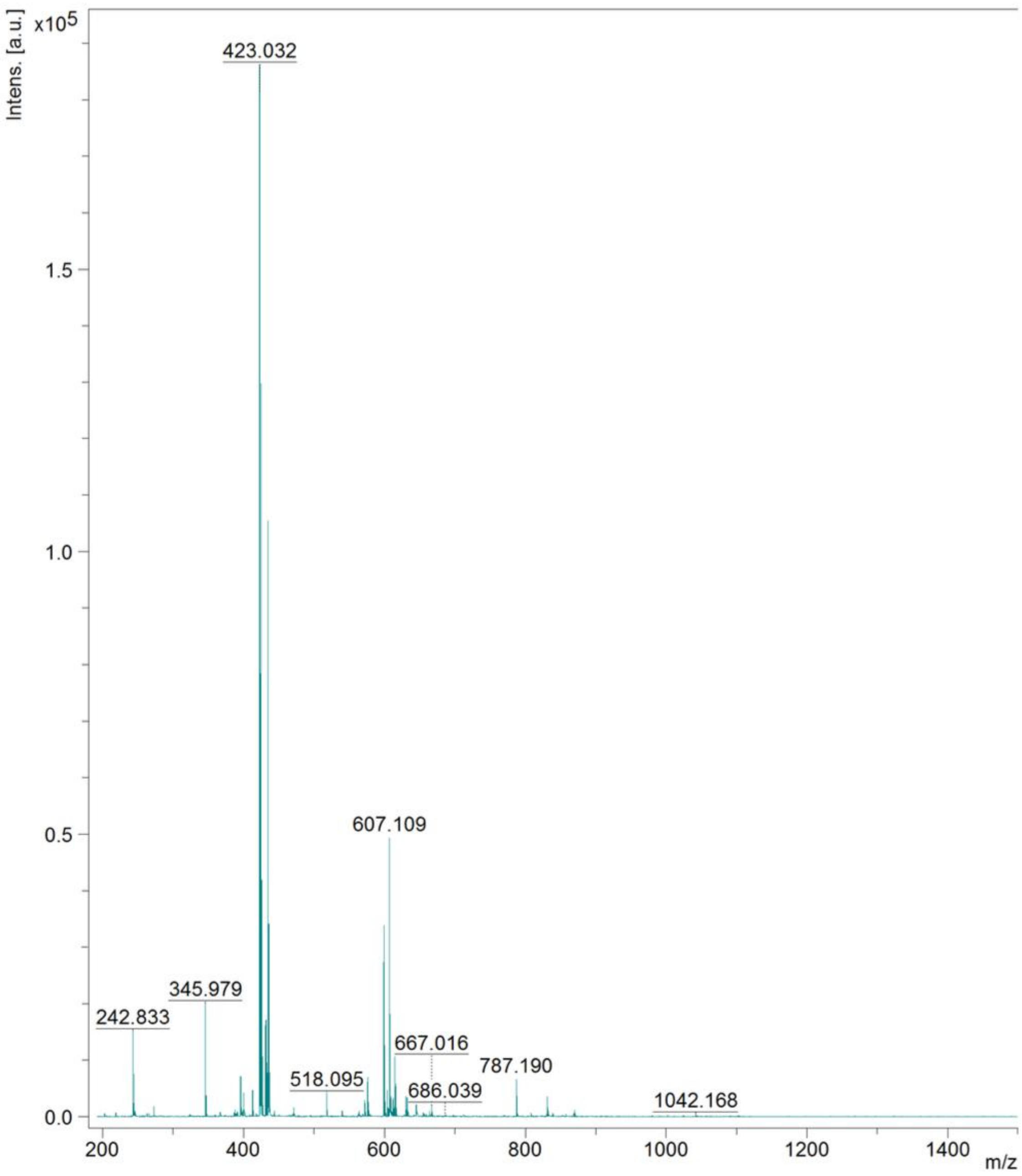

Figure S5. MALDI-TOF-MS spectrum of [VO(dipic)(phen)] · 3 H_2O (CCA matrix).

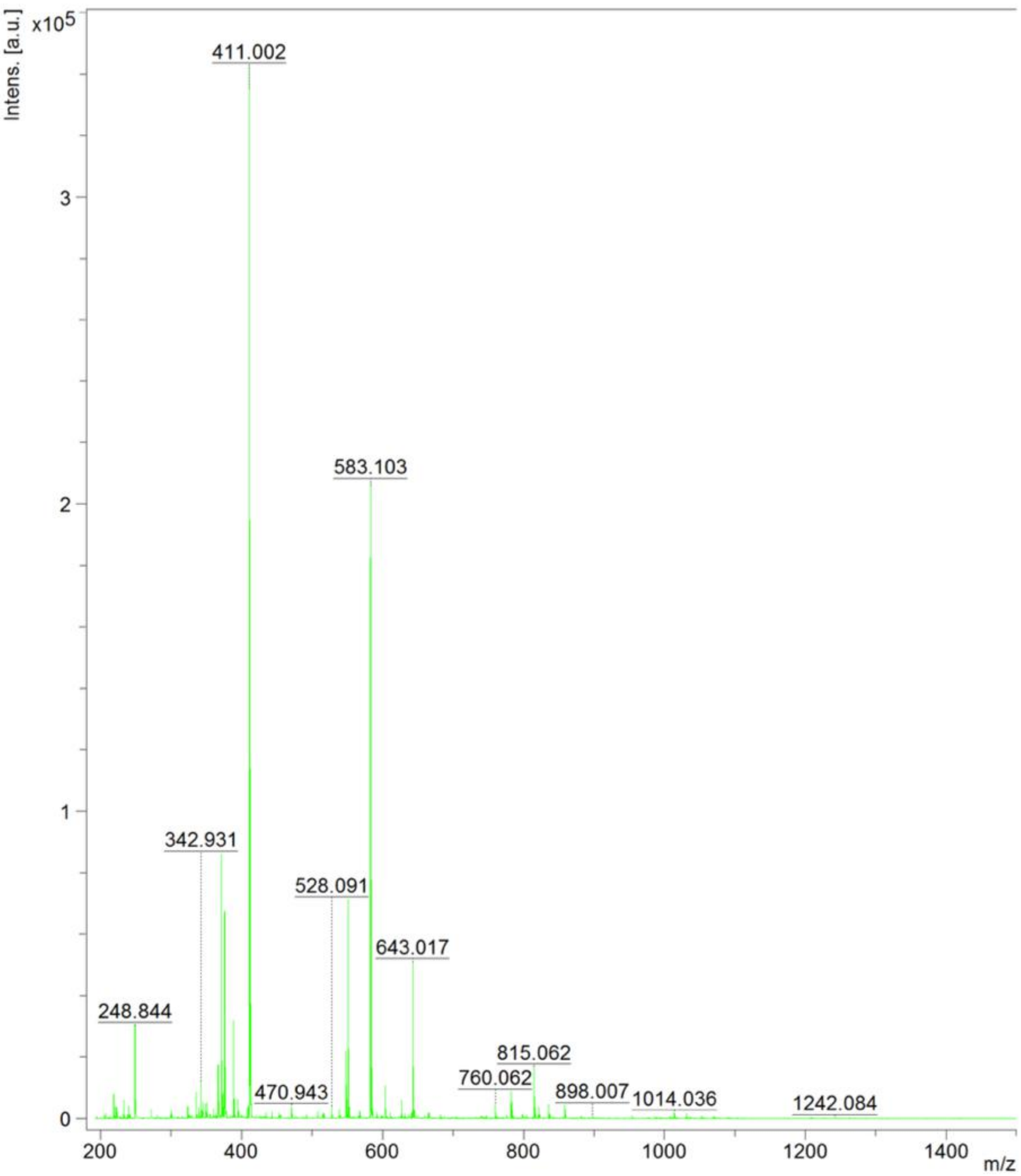

Figure S6. MALDI-TOF-MS spectrum of [VO(dipic)(bipy)] · H_2O (CCA matrix).

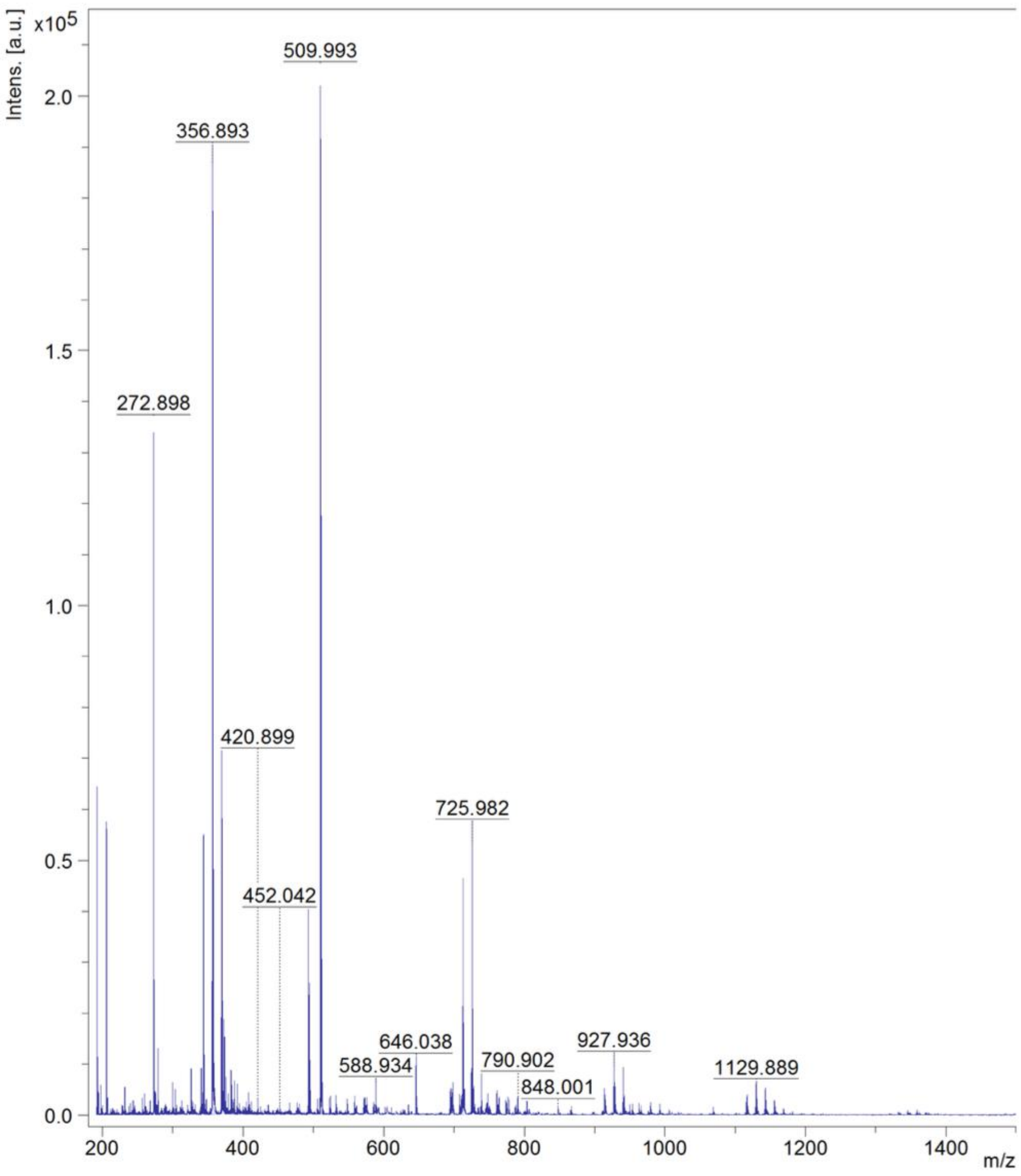

Figure S7. MALDI-TOF-MS spectrum of $[VO(dipic)(H_2O)_2] \cdot 2\ H_2O$ (DHB matrix).

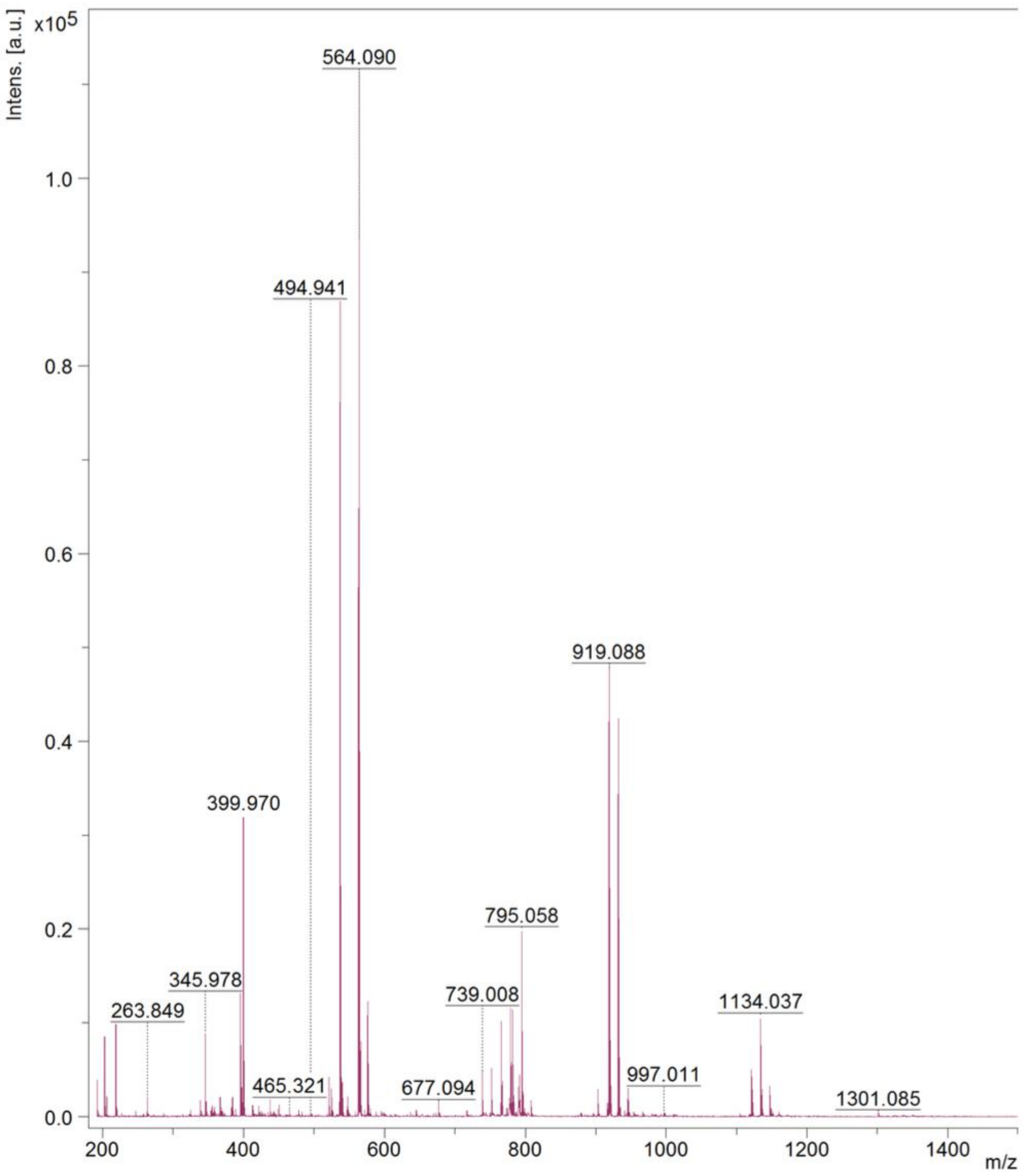

Figure S8. MALDI-TOF-MS spectrum of [VO(dipic)(phen)] · 3 H_2O (DHB matrix).

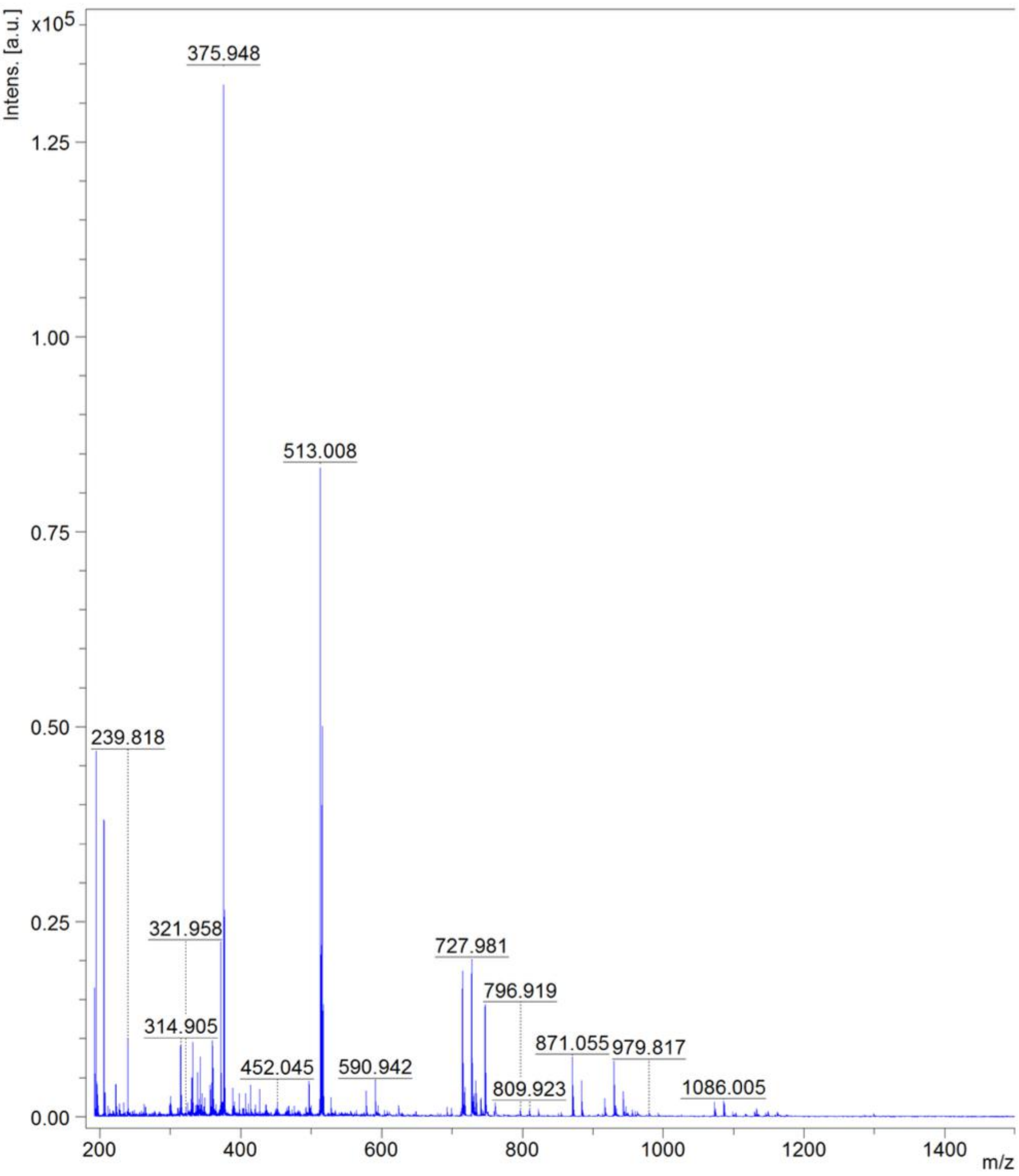

Figure S9. MALDI-TOF-MS spectrum of [VO(dipic)(bipy)] · H_2O (DHB matrix).

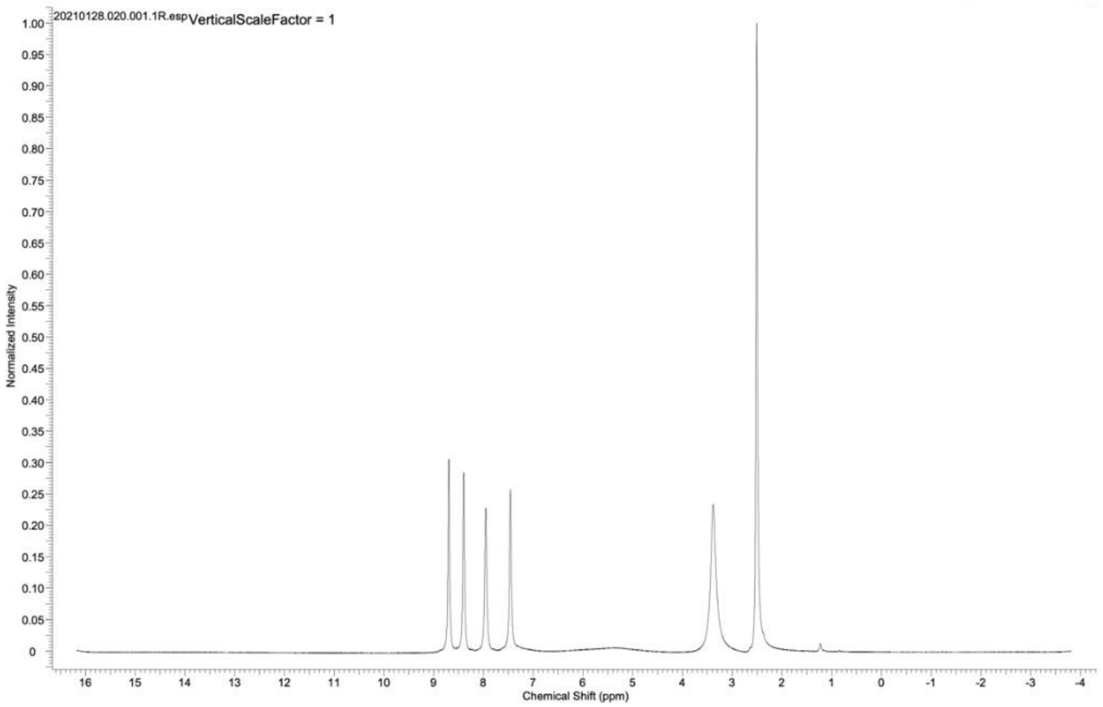

Figure S10. ^{1}H NMR spectrum of [VO(dipic)(bipy)] · H_2O.

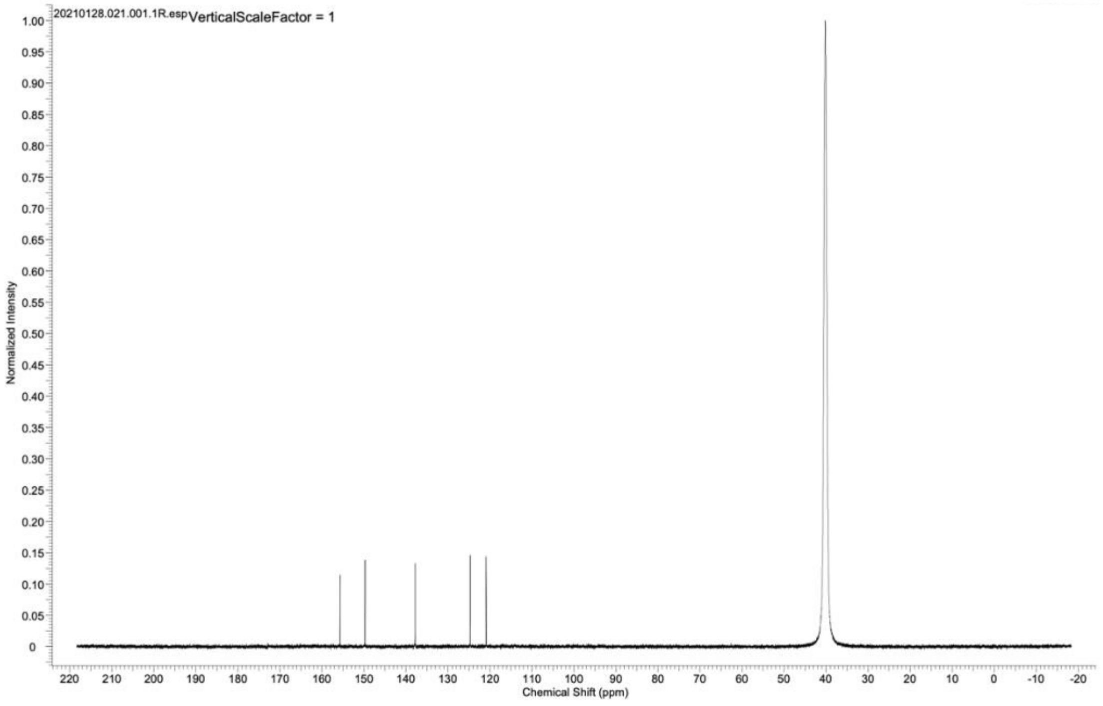

Figure S11. ^{13}C NMR spectrum of [VO(dipic)(bipy)] · H_2O.
